# Hidden Genetic Variability, Can the Olive Moth *Prays oleae* (Lepidoptera: Yponomeutidae or Praydidae?) be a Species’ Complex?

**DOI:** 10.3390/insects11040204

**Published:** 2020-03-26

**Authors:** Marlon Pazian, Tânia Nobre, Imen Blibech, Fernando T Rei

**Affiliations:** 1 MED—Mediterranean Institute for Agriculture, Environment and Development, Instituto de Investigação e Formação Avançada, Universidade de Évora, Pólo da Mitra, Ap. 94, 7006-554 Évora, Portugal; marlon.pazian@gmail.com (M.P.); frei@uevora.pt (F.T.R.); 2 Laboratory of research LR: Genetic Resources of the Olive Tree: Characterization, Promotion and Protection, Olive Tree Institute, Sousse 4061, Tunisia; imenblibech@gmail.com

**Keywords:** Prays oleae, olive moth, cryptic species, phylogenetic, population structure

## Abstract

*Prays oleae* is the second most important pest in Mediterranean olive groves, causing substantial damage on olive production. We used mitochondrial [cytochrome c oxidase subunit I (*COI*), and NADH dehydrogenase subunit 5 (*nad5*)] and nuclear [ribosomal protein S5 (*RpS5*)] amplicons to assess the population variability in five main olive producing regions from Tunisia, to support or dismiss the existence of two non-monophyletic groups within the species, as found within Portugal. Our phylogenetic analysis with cytochrome c oxidase subunit I (*COI*) indeed displayed two distinct and well-supported clades of *P. oleae*, which were corroborated by the haplotype network reconstructed with both mitochondrial and nuclear amplicons. We were also able to dismiss the hypothesis that one of the clades would not develop on olive fruits. No correlation was observed between clades differentiation and geographic distribution. The existence of cryptic species can impact on the management of agroecosystems and on the perception of how these moths responds to environmental changes.

## 1. Introduction

Global crop losses by insects are estimated to be 13% per annum despite the usage of multiple pesticides [1]. The insect order Lepidoptera includes many crop pests [2]; generally larval stages are crop destroyers that include defoliators, shoot/root borers, and seed predators causing significant agricultural losses. Some Lepidoptera species are known to cause damage to the olive trees, the major agro-ecosystem in the Mediterranean Basin, being the most significant in terms of impact the olive moth, *Prays oleae* (Bernard, 1978) (Lepidoptera, Yponomeutidae or Praydidae) [3,4].

The population dynamics of this moth is intrinsically dependent on the host-plant characteristics and development as its three yearly larval generations depend on the olive tree: (i) the phylophagous generation feeds on leaves; (ii) the anthophagous generation feeds on olive tree flowers and develops during the plant blooming; and (iii) the carpophagous generation feeds on olive fruits. The dietary preferences of adults are, however, poorly known but likely they feed on floral nectar and on a variety of other liquids similarly to most Lepidopteran adults [5]. Such a close and intricate connection between the olive moth and the olive tree should be reflected in the olive moth population structure and its co-evolutionary history. This co-history is already known and accepted for *Bactrocera oleae* (Diptera, Tephritidae) [6] whose larvae are monophagous, feeding exclusively on the tissue of olive fruits.

Recently, Nobre and co-workers [4] have questioned the species status of *P. oleae* as the reconstructed phylogeny based on the available data resolves this species as non-monophyletic. Moreover, the same study suggested the co-existence of two sympatric evolutionary lineages of morphologically cryptic olive moth populations. These two lineages overlapped geographically throughout the extensive sampling in Portugal [4]. Given this scenario, a local diversification could be hypothesized, particularly because the Iberian Peninsula is known as one of the most important Pleistocene glacial refugia in Europe. This claim is well supported by several lines of evidence, also for Lepidoptera species (e.g., two genetic lineages of *Aglaope infausta* with a likely differentiation center in Iberia [7]; the phylogeography of *Melitaea cinxia* shows the importance of the Iberian refugia in current structure [8], several refugia in the Iberian Peninsula have been inferred for the protected species *Graellsia isabellae* and its recognized plant host [9]). However, the currently available data on *Prays oleae* COI collected outside Portugal (one from Spain and three from Tunisia) suggest a similar pattern on the other side of the Mediterranean Sea [4].

For *Bactrocera oleae*, Segura and co-workers [10] found that the most southerly of the Mediterranean populations sampled (Tunisia) differed significantly from the remaining populations. However, Nardi and co-workers [6] suggested that those divergent Tunisian samples might in fact belong to a cluster of olive fruit flies in Central/Western Mediterranean area. More recently, Iberian and Italian *B. oleae* populations were shown to clearly split, giving rise to the existence of at least three well separated Mediterranean Basin populations: the Western Mediterranean, the Italian (including Greece and Western Turkey) and the Eastern Mediterranean clusters [11].

These findings triggered the present work [4,6,11]: we have sampled *P. oleae* in five important olive grove regions in Tunisia and proceed similarly with the previous approach done in Portugal [4], to search for population diversity and co-presence of the previously identified lineages.

## 2. Materials and Methods

### 2.1. Specimen Collection

**Figure 1:**
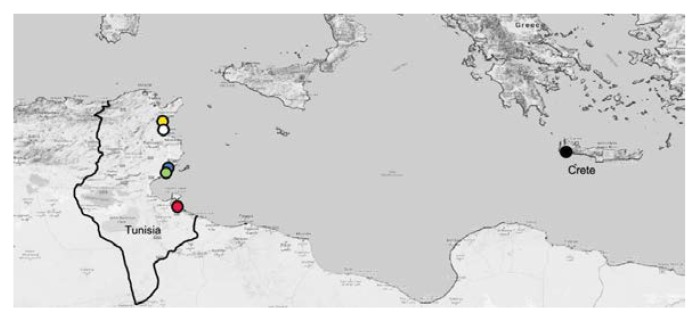
Main sampling regions of Tunisian and Greek *Prays oleae* utilized in this study. Red = Zazis, green = Chaffar, blue = Hajeb, white = Sidi Bouali, yellow = Bouficha and black = Crete.

Seventy-nine *P. oleae* specimens from Tunisia were sampled at five localities (Bouficha, Chaffar, Hajeb, Sidi Bouali, and Zarzis) and three specimens of *P. oleae* from Greece (that have emerged from olives collected in Crete island in 2019) (Figure 1). Tunisian specimens were sampled using commercial traps with specific pheromones (Biosani) during the year 2017.

### 2.2. DNA Extraction, PCR and Sequencing

Adult specimens were stored at −20 °C in 96% ethanol until DNA extraction. DNA extraction was performed from whole specimen body following extraction protocol described in [4]. DNA was eluted in 50 µL of sterile ultra-pure water and stored at −20 °C for posteriorly utilization in PCR reactions. Partial cytochrome c oxidase subunit I (*COI*), NADH dehydrogenase subunit 5 (*nad5*) and ribosomal protein S5 (*RpS5*) were amplified using primers:( 1) LCO1490 (5′- GGT CAA CAA ATC ATA AAG ATA TTG G -3’ and HCO2198 (5′- TAA ACT TCA GGG TGA CCA AAA AAT CA -3′) for a fragment of the *COI* gene [12]; (2) nad5_fw (5′- TTA TAT CCT TAG AAT AAA ATC C -3′) and nad5_rev (5′- TTA GGT TGA GAT GGT TTA GG -3′) for a fragment of the *nad5* gene [13] and (3) RpS5_f (5′- ATG GCN GAR GAR AAY TGG AAY GA -3′) and RpS5_r (5′- CGG TTR GAY TTR GCA ACA CG -3′) for a fragment of the *RpS5* gene [14].

PCR was carried out on a thermocycler with a final volume of 12.5 µL containing 0.25 µL dNTP (2 mM), 1.25 µL 10× Taq buffer, 0.25 µL each primer (10 mM), 0.7 µL MgCl_2_ (50 mM), 0.05 U/mL Taq DNA polymerase, 1 µL of the extracted DNA (10–20 ng), and ultrapure water. The PCR amplification conditions were as follows: 94 °C for 5 minutes, followed by 30 cycles of 94 °C for 30 seconds, specific annealing temperatures (55 °C for COI, nad5 52 °C and 53 °C RpS5) for 30 seconds and 72 °C for 1 minute and final extension cycle at 72 °C for 10 minutes. All PCR products were checked by electrophoresis (1% agarose gel). The PCR products, one per sample per amplicon, were purified with NZYGelpure Kit (NZYTech, Lda, Lisbon, Portugal) and sequencing was done commercially (Macrogen Inc. and EUROFINS, Madrid, Spain).

The assembly and editing of sequences were performed using GeneStudio program. Sequences were aligned with the Muscle algorithm in MEGA X [15] and were organized as haplotypes in DnaSP6 [16]. All haplotypes obtained in this study were submitted to the GenBank database (GenBank accession numbers (Supplementary Table S1): *RPS5* (from MT096181 to MT096259), *COI* (from MT096260 to MT096341) and *nad5* (from MT106246 to MT106324).

### 2.3. Phylogenetic Analysis

COI haplotypes observed in present study were combined with those available for *Prays* species on GenBank, and we performed a Bayesian phylogenetic reconstruction of *Prays oleae* using COI sequences in BEAST version v.4.2.8 [17]. Gamma site Model with 4 categories, and rate frequencies were estimated, other settings were kept as default (10 000 000 generations). BEAST results were analysed by Tracer v.1.6. Consensus tree was obtained using TreeAnnotator, first 10% trees were removed. To check phylogenetic reconstruction congruence, we performed Maximum Likelihood and Neighbor-Joining methods as implemented in MEGA X [15].

### 2.4. Variability and Population Structure

Mitochondrial and nuclear sequences (*COI*, *nad5* and *RpS5*) variability analysis was performed on DnaSP6 [16]. Genetic diversity (haplotype diversity [Hd] and nucleotide diversity [Pi]) was estimated, synonymous and non-synonymous sites were analysed together (as separation would imply a too low number of sites to yield reliable results [18]). Tajima’s D statistics was used to compare pairwise differences with the number of segregating sites [19]. ZnS statistics (the r2 squared allele frequency correlation [20]) was used to test linkage disequilibrium in all sampled fragments, based on the parsimony informational sites. The statistical support for the Zns and D of Tajima was evaluated by coalescent simulations with 10000 replicates in DnaSP6 [16], considering all segregating sites (α = 0.05). Analyses were performed for the three amplicons independently and concatenated. The reconstructed haplotype networks, both concatenated and in the three separate regions, were used to visualize the relationships among the sequences and were built using the TCS network (95% connection limit) in PopART [21].

## 3. Results

**Figure 2:**
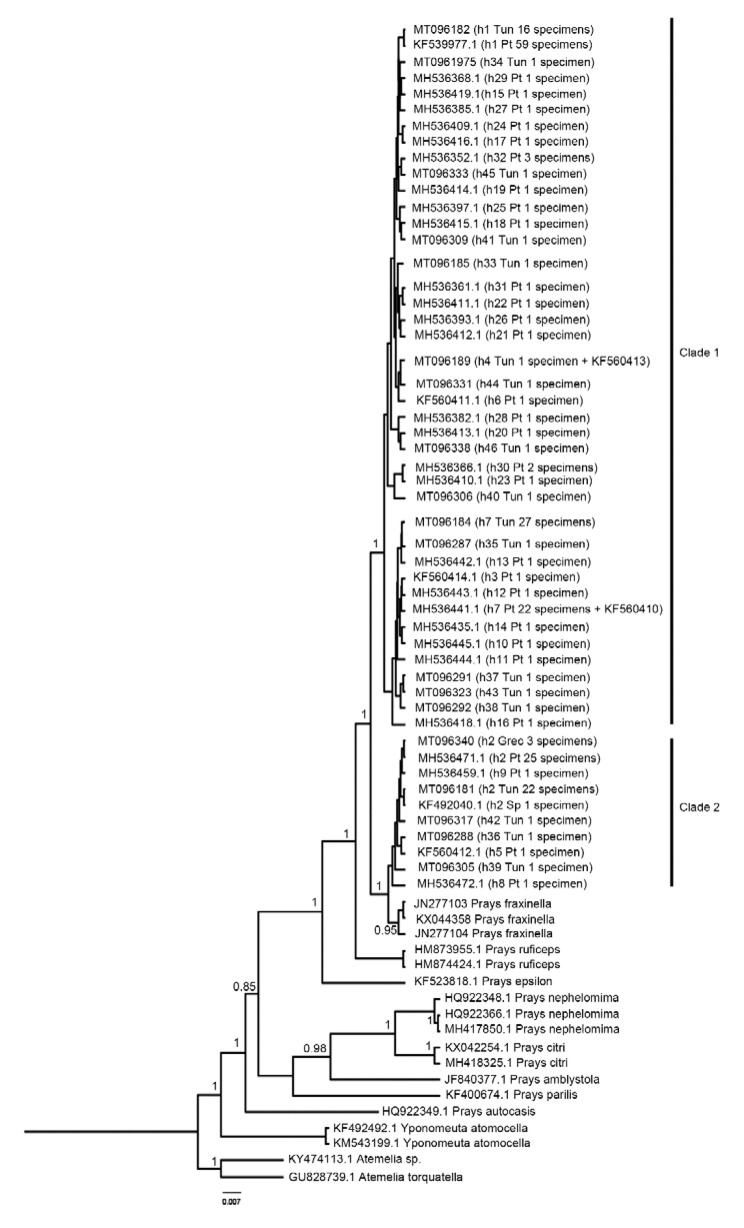
Cytochrome c oxidase subunit I (*COI*) Bayesian inference tree for *Prays oleae*. Nodes values are Bayesian posterior probabilities. Tree shows a clustering for polymorphic *Prays* (Clade 1 and Clade 2), suggesting the existence of cryptic species in genus *Prays*. Putative *P. oleae* samples collection country identified as: Tun—Tunisia; Pt—Portugal; Sp—Spain; Grec—Greece. *Prays fraxinella* JN277103—Italy; JN277104—Italy; KX044358—Norway. h represents haplotype. The tip labels read as follows: Genbank accession number (haplotype code; country code; number of specimens).

All Tunisian specimens were sequenced for the *COI*, *nad5* and *RpS5* amplicons. The specimens from Greece were only used for phylogenetic reconstruction and were only sequenced at the COI amplicon. Phylogenetic reconstruction of the *Prays* genus based on the cytochrome oxidase region (Figure 2) showed that *Prays oleae* has a non-monophyletic group (the Maximum-likelihood - Supplementary Figure S1- and Neighbor-joining -Supplementary Figure S2- methods corroborated this typology). Nuclear and mitochondrial DNA amplicons variability of Tunisian *P. oleae* group (*COI*, *nad5* and *RpS5*) is shown in Table 1.

**Figure 3:**
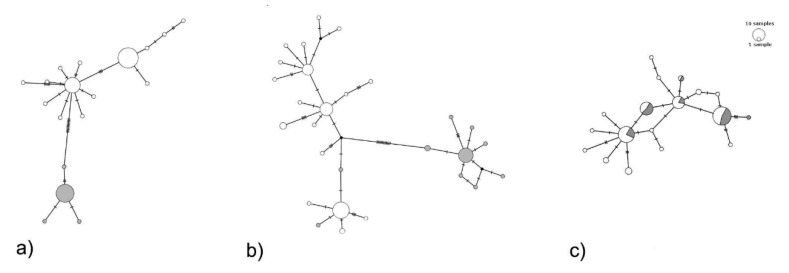
TCS haplotype network based on *COI* (**a**), *nad5* (**b**) and *RpS5* (**c**) amplicons of *Prays oleae* (circles, scaled to relative frequency of each haplotype in the data set). The white proportion in the circles correspond to the samples belonging to clade 1 and the grey part to clade 2 proportion according to COI phylogeny (Figure 2). *COI* (**a**) and *nad5* (**b**) networks show the occurrence of clade 1 and clade 2, while *RpS5* (**c**) might evolve too slowly to resolve the two clades.

Considering the complete dataset together, linkage disequilibrium was not detected, and Tajima statistics were not significant for all amplicons, suggesting that these DNA sequences evolved randomly (‘neutrality’) (Table 2). By analyzing clade 1 and clade 2 separately, Tajima D statistics was significant for both mitochondrial markers (Table 2). Tunisian *P. oleae* haplotype network (Figure 3a,b) consistently shows two groups separated by 11 mutational steps, for the haplotype network of both mtDNA amplicons, unlikely if the specimens represent a single species. The two lineages signal is faded when looking at the protein-coding nuclear gene region RpS5 amplicon (Figure 3c). No relation with sampling location was found (Supplementary Figure S3).

## 4. Discussion

*Prays oleae* reconstructed phylogeny resulted in two separate lineages with specimens forming a clade (clade 2) with *Prays fraxinella*, and a sister group clade (clade 1) with only *P. oleae* samples; this corroborates previous findings where we also found this distinct mitochondrial subdivision in the partial COI gene fragment [4]. The question on whether we are dealing with a single species or a group of cryptic species thus remains. On the raised question of whether these two differentiated lineages do have different ecological niches [4], we can now add to the discussion that we have observed specimens belonging to both clades emerging from olive fruits.

The fact that individuals of both lineages of putative *P. oleae* emerge from olive fruits is not surprising, but just a confirmation, as specimens of both studies were captured in olive groves. Several examples of phylogenetically related species complexes, hybrid specimen’s or cryptic species that present similar behavior and have no apparent phenotypic differences are reported [22,23,24,25,26]. The resource of molecular identification through species-specific markers help identify a given species quickly with a higher degree of accuracy (e.g. Barcode of Life) [27,28,29,30]. DNA barcoding is an extremely powerful tool and it was long established that the mitochondrial gene cytochrome c oxidase I (COI) is the core of the global identification system for animals [27]. However, the use of a mitochondrial amplicon only has also inherent limitations, including on hybrid identification for example [31,32]. Also, in phylogeography, mitochondrial DNA (mtDNA) has been extensively used due to its fast substitution rate, lack of recombination, small effective population size resulting in fast lineage sorting, and high sensitivity to demographic events (e.g. [33]). Even though the sole use of mtDNA reveals only a small part of the evolutionary history of a species, it has provided valuable phylogeographic data (e.g. [11,29,34,35]).

In the present study, the reconstruction of COI phylogeny supports the existence of two mitochondrial lineages in *P. oleae* (clade 1 and clade 2) coexisting in Tunisian and Portuguese olive groves. These data suggest the existence of a Central/Western Mediterranean olive moth group and not of a North-South Mediterranean differentiation. The only three available specimens from the Greek population belong to *Prays* clade 2, asking for further analyses to understand if a putative Central/Western—Eastern differentiation boundary exists and where it lies, and whether the Italian cluster (including Greece and Western Turkey) identified for *B. oleae* [11] is represented also in the *P. oleae* population structure. 

The haplotype network shows the two groups separated by several mutational steps, suggesting that specimens sampled unlikely represent a single species [36], given the strong structure among populations. The haplotype network of both mtDNA amplicons suggests the hypothesis that *P. oleae* may comprise more than one species. The nuclear region used, RpS5, likely evolves too slow to be able to discriminate the two clades signature. To be able to address the central question on whether this sub-division is uniquely mitochondrial or if it is also present at the nuclear level, several nuclear encoded marker(s) should be analysed. What seems clear from the mtDNA analyses is that there is a clear differentiation into two groups and ultimately these can correspond to two separate cryptic species [8,34,37]. Several studies demonstrate the effectiveness of using the COI gene fragment to discriminate known species and signal new ones, alone or in association with other genes fragments [26,30,34,37]. Despite the concerns of using this region alone [35], several studies show that it can reveal the existence of taxa with low divergence rate or recent radiation [26].

*Prays oleae*, although one of the most relevant pest species of olive groves, has been gained no or little attention from researchers dealing with population dynamics and structure. Our present results thus provide new insights, as they expand the geographic span of the available data and corroborate the existence of two different olive moth lineages highly differentiated and reconstructed as non-monophyletic on the basis of COI amplicon.

## 5. Conclusions

The *Prays oleae* paradigm will only be disentangled with a well-designed phylogenetic study comprising geographical meaningful samples of the two *P. oleae* lineages and *P. fraxinella*. From an agronomic perspective, the existence of cryptic pest species can have a high impact on agroecosystem management including in the perception of pest species reply to drastic environmental changes. From an evolutionary perspective, the impact of understanding population structure and eventual speciation is even higher by the fact that *Prays* belongs to the Yponomeutoidea superfamily which is thought to be one of the oldest among extant Ditrysian Lepidoptera [38].

## Figures and Tables

**Table 1:** Variability of the COI, nad5 and RpS5 amplicons analyzed, considering Tunisian *Prays oleae* dataset and partitioned by clade1 and clade 2.

Clades 1 and 2	COI	nad5	RpS5	Concatenated
Number of sequences	79	79	79	79
Number of sites (bp)	588	649	517	1754
Number of haplotypes	18	28	18	57
Polymorphic sites (S)	34	42	16	92
Parsimony informative	17	24	8	49
Total number of mutations	36	47	16	99
Haplotype diversity (Hd)	0.779	0.892	0.846	0.986
Aver. nucleotide diff. (k)	7.166	7.886	2.024	17.07
Nucleotide diversity (Pi)	0.012	0.012	0.003	0.009
**Clade 1**	**COI**	**nad5**	**RpS5**	**Concatenated**
Number of sequences	55	55	55	55
Number of sites (bp)	588	649	517	1754
Number of haplotypes	14	18	17	45
Polymorphic sites (S)	21	29	14	83
Parsimony informative	5	13	8	46
Total number of mutations	21	33	14	88
Haplotype diversity (Hd)	0.683	0.838	0.874	0.987
Aver. nucleotide diff. (k)	2.261	3.521	2.149	9.237
Nucleotide diversity (Pi)	0.003	0.005	0.004	0.005
**Clade 2**	**COI**	**nad5**	**RpS5**	**Concatenated**
Number of sequences	24	24	24	24
Number of sites (bp)	588	649	517	1754
Number of haplotypes	4	10	6	14
Polymorphic sites (S)	4	25	7	34
Parsimony informative	0	16	3	18
Total number of mutations	4	25	7	34
Haplotype diversity (Hd)	0.239	0.667	0.757	0.92
Aver. nucleotide diff. (k)	0.333	3.431	1.648	5.17
Nucleotide diversity (Pi)	0	0.004	0.003	0.002

**Table 2:** Inferences on Tunisian population variability based on Tajima’s D, the site frequency spectrum (SFS) of mutations; and ZnS, the statistical association among those (linkage disequilibrium).

Clades 1 and 2	ZnS	Significance	Tajima’s D	Significance
COI	0.17	*p* = 0.96; [0.04, 0.17]	−0.05	*p* = 0.49; [−1.32, 1.33]
nad5	0.11	*p* = 0.78; [0.04, 0.17]	−0.55	*p* = 0.24; [−1.32, 1.42]
RpS5	0.04	*p* = 0.20; [0.00, 0.25]	−1.08	*p* = 0.10; [−1.52, 1.55]
Concatenated	0.08	*p* = 0.53; [0.04, 0.15]	−0.50	*p* = 0.23; [−1.28, 1.23]
**Clade 1**	**ZnS**	**Significance**	**Tajima’s D**	**Significance**
COI	0.09	*p* = 0.47; [0.01, 0.26]	−1.60	*p* = 0.009; [−1.45, 1.51]
nad5	0.07	*p* = 0.23; [0.03, 0.23]	−1.69	*p* = 0.005; [−1.46, 1.63]
RpS5	0.04	*p* = 0.17; [0.01, 0.29]	−0.89	*p* = 0.14; [−1.57, 1.72]
Concatenated	0.10	*p* = 0.59; [0.05, 0.18]	−1.80	*p* = 0.00; [−1.30, 1.27]
**Clade 2**	**ZnS**	**Significance**	**Tajima’s D**	**Significance**
COI	0.17	*p* = 0.70; [0.03, 0.60]	−1.88	*p* = 0.00; [−1.51, 1.71]
nad5	0.31	*p* = 0.95; [0.05, 0.35]	−1.81	*p* = 0.01; [−1.56, 1.38]
RpS5	0.21	*p* = 0.77; [0.00, 0.45]	−0.37	*p* = 0.34; [−1.57, 1.67]
Concatenated	0.20	*p* = 0.81; [0.06, 0.30]	−1.64	*p* = 0.01; [−1.51, 1.39]

## Supplementary Materials

The following are available online at https://www.mdpi.com/2075-4450/11/4/204/s1, Table S1. Data on the specimen and sampling coordinates of *Prays oleae* collected in Tunisia and Greece. GenBank accession numbers are given per sample; Figure S1. Maximum likelihood inference for *Prays oleae* samples using Tamura-Nei model. Nodes values are bootstrap statistic. The tree with the highest log likelihood is shown. The percentage of trees in which the associated taxa clustered together is shown next to the branches. Initial tree(s) for the heuristic search were obtained automatically by applying Neighbor-Join and BioNJ algorithms to a matrix of pairwise distances estimated using the Maximum Composite Likelihood (MCL) approach, and then selecting the topology with superior log likelihood value. The tree is drawn to scale, with branch lengths measured in the number of substitutions per site. Evolutionary analyses were conducted in MEGA X. h = haplotype, Grec = Greece, Pt = Portugal, Sp = Spain, Tun = Tunisia; Figure S2: Neighbor joining inference for *Prays oleae* samples. The percentage of replicate trees in which the associated taxa clustered together in the bootstrap test (10,000 replicates) are shown next to the branches. All positions containing gaps and missing data were eliminated (complete deletion). Evolutionary analyses were conducted in MEGA X. h = haplotype, Grec = Greece, Pt = Portugal, Sp = Spain, Tun = Tunisia. Figure S3. TCS haplotype network based on COI (a), nad5 (b) and RpS5 (c) amplicons of *Prays oleae* (circles, scaled to relative frequency of each haplotype in the data set); Note the absence of correlation between sampling locality and group (clade1 or clade 2; Figure 3).
