# Supplementary material for: Hidden Genetic Variability, Can the Olive Moth Prays oleae (Lepidoptera: Yponomeutidae or Praydidae?) be a Species’ Complex?
Source: Insects. 2020 Mar 26;11(4):204. doi: 10.3390/insects11040204 (PMC7240722; doi:10.3390/insects11040204)
Supplement: Supplementary file 1 [file insects-11-00204-s001.pdf]

Hidden genetic variability, can the olive moth *Prays oleae* (Lepidoptera: Yponomeutidae or Praydidae?) be a 'species' complex?

Pazian, M; Nobre, T.*; Blibech, I; Rei, F.T.

MED – Mediterranean Institute for Agriculture, Environment and Development, Instituto de Investigação e Formação Avançada, Universidade de Évora, Pólo da Mitra, Ap. 94, 7006-554 Évora, Portugal.

*Corresponding author: tnobre@uevora.pt

Key words: *Prays oleae*, olive moth, cryptic species, phylogenetic, population structure

Table S1. Data on the specimen and sampling coordinates of *Prays oleae* collected in Tunisia and Greece. GenBank accession numbers are given per sample. N/S = no sampled amplicon.

Lab voucher	Locality	COI phylogeny (Fig.2)	Genbank accession number			Coordinates
			COI	nad5	RpS5	
B12	Bouficha	clade 2	MT096260	MT106246	MT096181	36°15'36.0"N 10°26'24.0"E
B13	Bouficha	clade 1	MT096261	MT106247	MT096182	36°15'36.0"N 10°26'24.0"E
B14	Bouficha	clade 1	MT096262	MT106248	MT096183	36°15'36.0"N 10°26'24.0"E
B16	Bouficha	clade 1	MT096263	MT106249	MT096184	36°15'36.0"N 10°26'24.0"E
B21	Bouficha	clade 1	MT096264	MT106250	MT096185	36°15'36.0"N 10°26'24.0"E
B22	Bouficha	clade 1	MT096265	MT106251	MT096186	36°15'36.0"N 10°26'24.0"E
B23	Bouficha	clade 1	MT096266	MT106252	MT096187	36°15'36.0"N 10°26'24.0"E
B24	Bouficha	clade 1	MT096267	MT106253	MT096188	36°15'36.0"N 10°26'24.0"E
B25	Bouficha	clade 1	MT096268	MT106254	MT096189	36°15'36.0"N 10°26'24.0"E
B31	Bouficha	clade 2	MT096269	MT106255	MT096190	36°15'36.0"N 10°26'24.0"E
B32	Bouficha	clade 1	MT096270	MT106256	MT096191	36°15'36.0"N 10°26'24.0"E
B33	Bouficha	clade 2	MT096271	MT106257	MT096192	36°15'36.0"N 10°26'24.0"E
B34	Bouficha	clade 1	MT096272	MT106258	MT096193	36°15'36.0"N 10°26'24.0"E
B35	Bouficha	clade 1	MT096273	MT106259	MT096194	36°15'36.0"N 10°26'24.0"E
B36	Bouficha	clade 1	MT096274	MT106260	MT096195	36°15'36.0"N 10°26'24.0"E
C14	Chaffar	clade 1	MT096275	MT106261	MT096196	34°33'36.0"N 10°33'36.0"E
C15	Chaffar	clade 1	MT096276	MT106262	MT096197	34°33'36.0"N 10°33'36.0"E
C17	Chaffar	clade 1	MT096277	MT106263	MT096198	34°33'36.0"N 10°33'36.0"E
C210	Chaffar	clade 1	MT096278	MT106264	MT096199	34°33'36.0"N 10°33'36.0"E
C23	Chaffar	clade 1	MT096279	MT106265	MT096200	34°33'36.0"N 10°33'36.0"E
C25	Chaffar	clade 2	MT096280	MT106266	MT096201	34°33'36.0"N 10°33'36.0"E
C26	Chaffar	clade 2	MT096281	MT106267	MT096202	34°33'36.0"N 10°33'36.0"E
C27	Chaffar	clade 1	MT096282	MT106268	MT096203	34°33'36.0"N 10°33'36.0"E
C28	Chaffar	clade 2	MT096283	MT106269	MT096204	34°33'36.0"N 10°33'36.0"E
C29	Chaffar	clade 2	MT096284	MT106270	MT096205	34°33'36.0"N 10°33'36.0"E
C31	Chaffar	clade 1	MT096285	MT106271	MT096206	34°33'36.0"N 10°33'36.0"E
C35	Chaffar	clade 1	MT096286	MT106272	MT096207	34°33'36.0"N 10°33'36.0"E
C36	Chaffar	clade 1	MT096287	MT106273	MT096208	34°33'36.0"N 10°33'36.0"E
C37	Chaffar	clade 2	MT096288	MT106274	MT096209	34°33'36.0"N 10°33'36.0"E
C38	Chaffar	clade 1	MT096289	MT106275	MT096210	34°33'36.0"N 10°33'36.0"E
C39	Chaffar	clade 1	MT096290	MT106276	MT096211	34°33'36.0"N 10°33'36.0"E
H12	Hajeb	clade 1	MT096291	MT106277	MT096212	34°43'12.0"N 10°37'48.0"E
H16	Hajeb	clade 1	MT096292	MT106278	MT096213	34°43'12.0"N 10°37'48.0"E
H17	Hajeb	clade 1	MT096293	MT106279	MT096214	34°43'12.0"N 10°37'48.0"E
H19	Hajeb	clade 1	MT096294	MT106280	MT096215	34°43'12.0"N 10°37'48.0"E
H21	Hajeb	clade 2	MT096295	MT106281	MT096216	34°43'12.0"N 10°37'48.0"E
H23	Hajeb	clade 1	MT096296	MT106282	MT096217	34°43'12.0"N 10°37'48.0"E
H24	Hajeb	clade 1	MT096297	MT106283	MT096218	34°43'12.0"N 10°37'48.0"E
H25	Hajeb	clade 1	MT096298	MT106284	MT096219	34°43'12.0"N 10°37'48.0"E
H26	Hajeb	clade 2	MT096299	MT106285	MT096220	34°43'12.0"N 10°37'48.0"E

Lab voucher	Locality	COI phylogeny (Fig.2)	Genbank accession number			Coordinates
			COI	nad5	RpS5	
H28	Hajeb	clade 1	MT096300	MT106286	MT096221	34°43'12.0"N 10°37'48.0"E
H31	Hajeb	clade 2	MT096301	MT106287	MT096222	34°43'12.0"N 10°37'48.0"E
H32	Hajeb	clade 1	MT096302	MT106288	MT096223	34°43'12.0"N 10°37'48.0"E
H33	Hajeb	clade 1	MT096303	MT106289	MT096224	34°43'12.0"N 10°37'48.0"E
H36	Hajeb	clade 1	MT096304	MT106290	MT096225	34°43'12.0"N 10°37'48.0"E
H38	Hajeb	clade 2	MT096305	MT106291	MT096226	34°43'12.0"N 10°37'48.0"E
S11	Sidi Bouali	clade 1	MT096306	MT106292	MT096227	35°58'48.0"N 10°28'48.0"E
S12	Sidi Bouali	clade 1	MT096307	MT106293	MT096228	35°58'48.0"N 10°28'48.0"E
S13	Sidi Bouali	clade 1	MT096308	MT106294	MT096229	35°58'48.0"N 10°28'48.0"E
S14	Sidi Bouali	clade 1	MT096309	MT106295	MT096230	35°58'48.0"N 10°28'48.0"E
S15	Sidi Bouali	clade 1	MT096310	MT106296	MT096231	35°58'48.0"N 10°28'48.0"E
S16	Sidi Bouali	clade 1	MT096311	MT106297	MT096232	35°58'48.0"N 10°28'48.0"E
S21	Sidi Bouali	clade 1	MT096312	MT106298	MT096233	35°58'48.0"N 10°28'48.0"E
S22	Sidi Bouali	clade 2	MT096313	MT106299	MT096234	35°58'48.0"N 10°28'48.0"E
S23	Sidi Bouali	clade 2	MT096314	MT106300	MT096235	35°58'48.0"N 10°28'48.0"E
S24	Sidi Bouali	clade 2	MT096315	MT106301	MT096236	35°58'48.0"N 10°28'48.0"E
S25	Sidi Bouali	clade 2	MT096316	MT106302	MT096237	35°58'48.0"N 10°28'48.0"E
S31	Sidi Bouali	clade 2	MT096317	MT106303	MT096238	35°58'48.0"N 10°28'48.0"E
S32	Sidi Bouali	clade 1	MT096318	MT106304	MT096239	35°58'48.0"N 10°28'48.0"E
S33	Sidi Bouali	clade 1	MT096319	MT106305	MT096240	35°58'48.0"N 10°28'48.0"E
S36	Sidi Bouali	clade 1	MT096320	MT106306	MT096241	35°58'48.0"N 10°28'48.0"E
Z11	Zarzis	clade 2	MT096321	MT106307	MT096242	33°25'48.0"N 10°56'24.0"E
Z12	Zarzis	clade 2	MT096322	MT106308	MT096243	33°25'48.0"N 10°56'24.0"E
Z13	Zarzis	clade 1	MT096323	MT106309	MT096244	33°25'48.0"N 10°56'24.0"E
Z15	Zarzis	clade 1	MT096324	MT106310	MT096245	33°25'48.0"N 10°56'24.0"E
Z16	Zarzis	clade 2	MT096325	MT106311	MT096246	33°25'48.0"N 10°56'24.0"E
Z17	Zarzis	clade 1	MT096326	MT106312	MT096247	33°25'48.0"N 10°56'24.0"E
Z21	Zarzis	clade 2	MT096327	MT106313	MT096248	33°25'48.0"N 10°56'24.0"E
Z22	Zarzis	clade 1	MT096328	MT106314	MT096249	33°25'48.0"N 10°56'24.0"E
Z23	Zarzis	clade 1	MT096329	MT106315	MT096250	33°25'48.0"N 10°56'24.0"E
Z24	Zarzis	clade 2	MT096330	MT106316	MT096251	33°25'48.0"N 10°56'24.0"E
Z25	Zarzis	clade 1	MT096331	MT106317	MT096252	33°25'48.0"N 10°56'24.0"E
Z26	Zarzis	clade 1	MT096332	MT106318	MT096253	33°25'48.0"N 10°56'24.0"E
Z27	Zarzis	clade 1	MT096333	MT106319	MT096254	33°25'48.0"N 10°56'24.0"E
Z31	Zarzis	clade 2	MT096334	MT106320	MT096255	33°25'48.0"N 10°56'24.0"E
Z32	Zarzis	clade 1	MT096335	MT106321	MT096256	33°25'48.0"N 10°56'24.0"E
Z33	Zarzis	clade 1	MT096336	MT106322	MT096257	33°25'48.0"N 10°56'24.0"E
Z34	Zarzis	clade 2	MT096337	MT106323	MT096258	33°25'48.0"N 10°56'24.0"E
Z35	Zarzis	clade 1	MT096338	MT106324	MT096259	33°25'48.0"N 10°56'24.0"E
Gre16	Crete	clade 2	MT096339	N/S	N/S	35°15'00.0"N 23°47'24.0"E
Gre17	Crete	clade 2	MT096340	N/S	N/S	35°15'00.0"N 23°47'24.0"E
Gre19	Crete	clade 2	MT096341	N/S	N/S	35°15'00.0"N 23°47'24.0"E

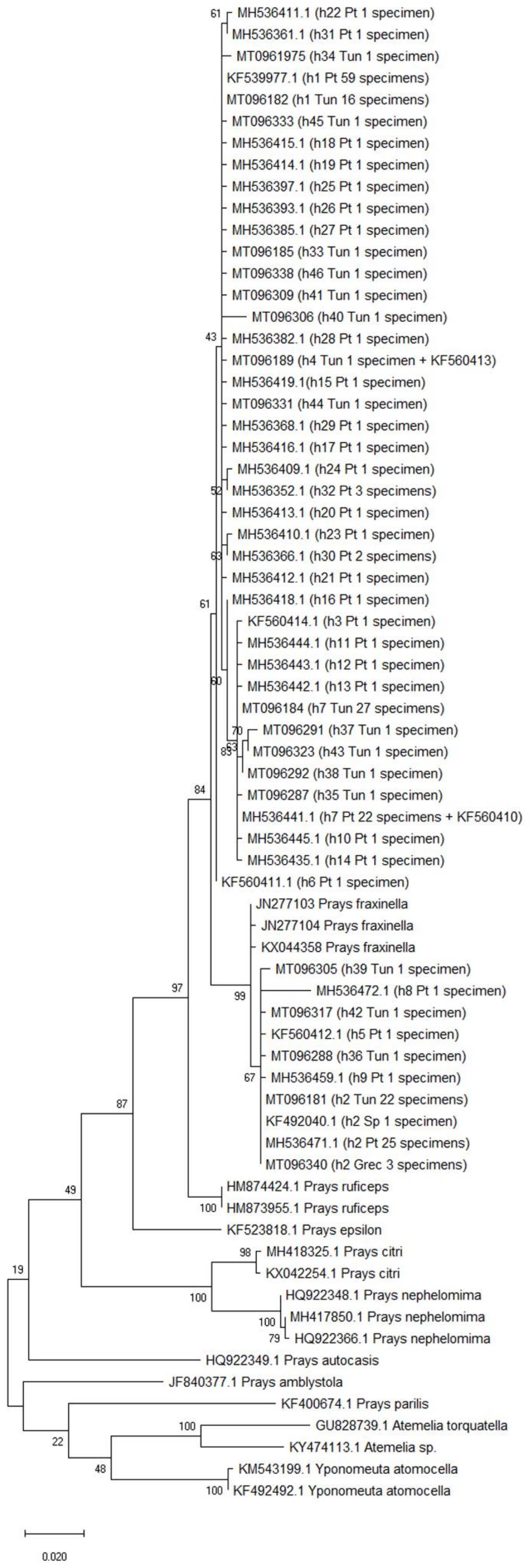

Figure S1. Maximum likelihood inference of *Prays oleae* samples using Tamura-Nei model. Nodes values are bootstrap statistic. The tree with the highest log likelihood is shown. The percentage of trees in which the associated taxa clustered together is shown next to the branches. Initial tree(s) for the heuristic search were obtained automatically by applying Neighbor-Join and BioNJ algorithms to a matrix of pairwise distances estimated using the Maximum Composite Likelihood (MCL) approach, and then selecting the topology with superior log likelihood value. The tree is drawn to scale, with branch lengths measured in the number of substitutions per site. Evolutionary analyses were conducted in MEGA X. h= haplotype, Grec= Greece, Pt= Portugal, Sp= Spain, Tun= Tunisia

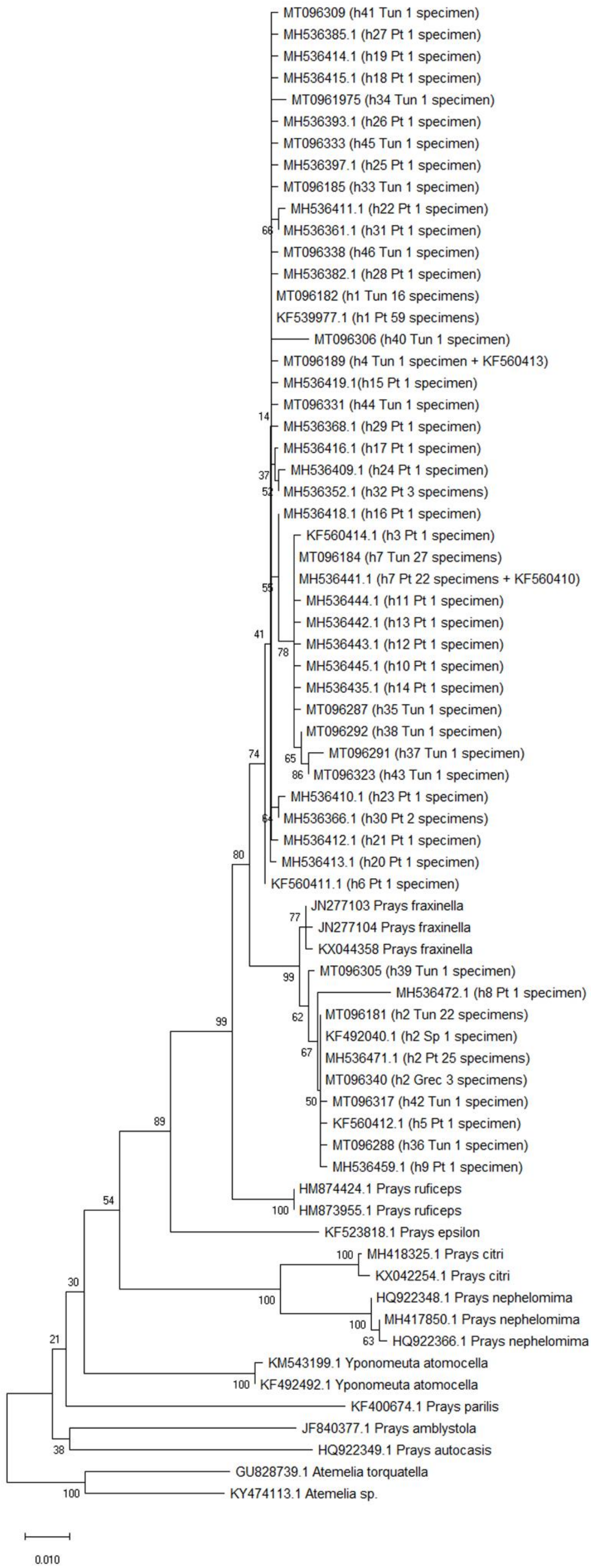

Figure S2: Neighbor joining inference for *Prays oleae* samples. The percentage of replicate trees in which the associated taxa clustered together in the bootstrap test (10000 replicates) are shown next to the branches. The evolutionary distances were computed using the Kimura 2-parameter method All positions containing gaps and missing data were eliminated (complete deletion). Evolutionary analyses were conducted in MEGA X. h= haplotype, Grec= Greece, Pt= Portugal, Sp= Spain, Tun= Tunisia.

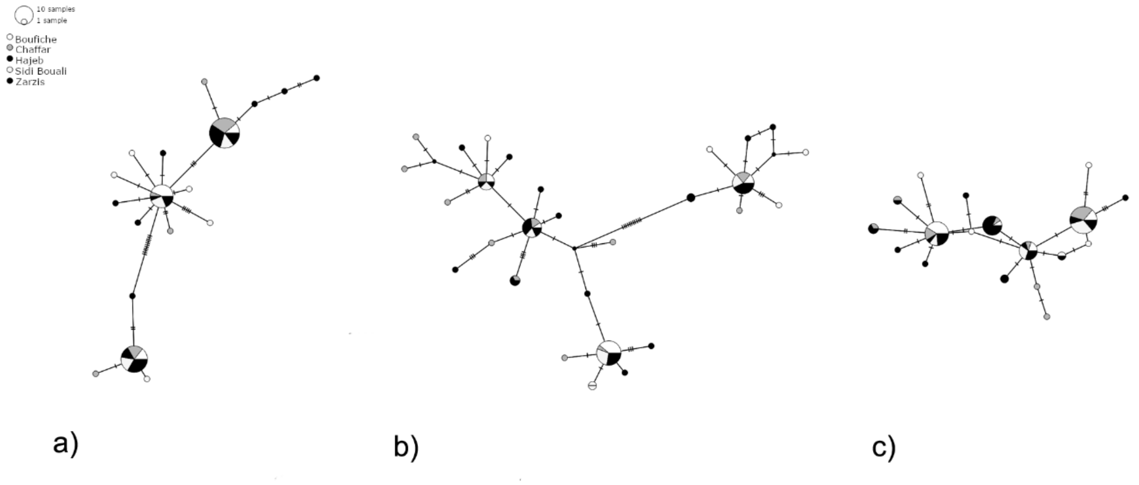

Figure S3. TCS haplotype network based on COI (a), nad5 (b) and RpS5 (c) amplicons of *Prays oleae* (circles, scaled to relative frequency of each haplotype in the data set); Note absence of correlation between sampling locality and group (clade1 or clade 2; Figure 3).
